# Correlating quantitative real-time PCR to rapid diagnostic test and RNA transcript expression in isolated gametocytemia and asexual parasitemia of *Plasmodium falciparum* malaria

**DOI:** 10.1186/s40794-015-0008-3

**Published:** 2015-09-08

**Authors:** Rachel Lau, Melissa Phuong, Filip Ralevski, Andrea K. Boggild

**Affiliations:** 1 Public Health Ontario Laboratories, Public Health Ontario, Toronto, Canada; 2 Faculty of Health Sciences, McMaster University, Hamilton, Canada; 3 Tropical Disease Unit, Division of Infectious Diseases, UHN-Toronto General Hospital, 200 Elizabeth Street, 13EN-218, Toronto, ON M5G 2C4 Canada; 4 Department of Medicine, University of Toronto, Toronto, Canada

## Abstract

**Background:**

At present, only microscopic examination of stained thick and thin blood smears for malaria can differentiate clinically relevant asexual parasitemia from clinically irrelevant isolated gametocytemia. Microscopy is time consuming, labour intensive, and requires significant technical expertise to perform. Simple and rapid tests that can distinguish asexual from isolated sexual parasitemia are needed.

**Methods:**

To determine if parasitemia and cycle threshold (C_T)_ values on *Plasmodium* genus and *P. falciparum*-specific quantitative polymerase chain reaction (qPCR) assays correlate to positivity of rapid diagnostic test (RDT), and 18S rRNA gene copy number, we analyzed blood samples from Ontario patients with isolated *P. falciparum* gametocytemia or asexual stages. RNA transcripts were evaluated to determine whether there is correlation of expression to different life cycle stages of *P. falciparum*.

**Results:**

45 specimens containing isolated *P. falciparum* gametocytes, and 40 specimens containing isolated asexual stages by microscopy were identified and analyzed. By RDT, 40 of 45 (88.9 %) isolated gametocytemia specimens and 40 of 40 (100 %) asexual-stage specimens were positive for *Plasmodium falciparum*-specific histidine rich protein-2 (HRP-2). Fourteen of 45 (31.1 %) isolated gametocytemia specimens, and 36 of 40 (90 %) asexual-stage specimens were positive for *Plasmodium* genus aldolase T2 band. Positivity of the aldolase T2 band was associated with lower mean *Plasmodium* genus and *P. falciparum-*specific C_T_ values, and to higher mean 18S rRNA gene copy by qPCR for both isolated gametocytemia and asexual-stage specimens. There was also a negative correlation of asexual parasitemia to both C_T_ values, and positive correlation to 18S rRNA gene copy number. Analysis of asexual stage-specific erythrocyte binding antigen (eba-175) transcripts on 25 isolated gametocytemia and 20 asexual-stage specimens gave a positive predictive value of 62.5 % and negative predictive value of 100 % for asexual parasitemia. Thus, an absence of eba-175 transcripts excluded the presence of asexual (clinically relevant) parasitemia.

**Conclusions:**

Positivity of the aldolase T2 band of BinaxNow RDT correlated to higher parasite load in both isolated gametocytemia and asexual-stage specimens. Asexual stage-specific eba-175 RNA transcript expression provided reasonable negative predictive value for exclusion of asexual parasitemia in clinical samples, but was present in both isolated gametocytemia and asexual stage specimens.

## Background

Prompt and accurate diagnosis of malaria is essential to limiting morbidity and mortality due to this imported parasitic infection [1]. Several tests are employed in the clinical parasitology laboratory to diagnose malaria, and these commonly include thick and thin Giemsa-stained blood smears for direct examination of parasitized erythrocytes; rapid antigen detection via immunochromatographic test; and amplification of parasite genomic material via PCR using qualitative or quantitative assays. Each of these laboratory tests has its own advantages and limitations, and unique performance characteristics [2, 3]. However, only direct examination of blood smears can, to date, reliably differentiate asexual parasitemia, which is clinically relevant, from sexual parasitemia or gametocytemia, which is clinically irrelevant meaning that these stages do not require treatment for resolution of malaria. Isolated gametocytemia is relevant from a public health perspective, as gametocytes are infectious to biting *Anopheles* mosquitoes present in parts of North America. Although rapid diagnostic (antigen) tests (RDTs) are a useful adjunct to direct examination of blood smears, they are unable to distinguish current from past infection due to antigen persistence following infection [4, 5], and the presence of gametocytes [6]. Similarly, commonly employed PCR assays amplify conserved targets, such as 18S, present in both asexual and sexual stage parasites [7, 8].

Understanding a patient’s parasitemia status over time will guide the course of treatment. If a patient remains symptomatic while on malaria treatment due to the presence of asexual stages (and potentially increasing asexual parasitemia), then this may reflect drug resistance or clinical failure. Conversely, if that patient remains symptomatic with fever but only sexual parasitemia is noted, then an alternate explanation for ongoing or recurrent symptoms would be pursued, rather than invoking drug-related issues. As RDT will remain positive for weeks after treatment [4, 5], and expertise in microscopy at hospital-based hematology laboratories is becoming increasingly scarce, PCR is playing an increasingly important and relevant role in the clinical management of malaria patients. Thus, identifying non-microscopy based tests with equal ability to identify stage and quantitation of parasites as microscopy is essential as our understanding of a patient’s ongoing parasitemia stage has direct clinical relevance.

The extent to which quantitative real time PCR results correlate to level of positivity on rapid diagnostic tests (RDTs) or C_T_ values when isolated gametocytemia is present in clinical samples is unknown, but could be useful for delineating laboratory “markers” or flags of isolated sexual parasitemia (gametocytemia) when smear results are unavailable or non-diagnostic for either quantitation or species identification. We sought to determine if parasitemia and C_T_ values on *Plasmodium* genus and *P. falciparum*-specific PCR assays and 18S rRNA gene copy number by quantitative real time PCR correlate to positivity of RDT in clinical samples from Ontario containing isolated *P. falciparum* gametocytemia or asexual stages by expert microscopy. As well, we evaluated RNA transcript markers that may differentiate sexual from asexual stages of *P. falciparum*.

## Methods

### Clinical samples

Samples included in this analysis were 45 biobanked malaria specimens with isolated *P. falciparum* gametocytemia noted at microscopy, and a random sampling of 10 each of specimens with the following ranges of *P. falciparum* asexual parasitemia (rings, trophozoites, and/or schizont stages) quantitated by expert microscopy: <0.1; 0.1–1.0; 1.0–10.0; and >10.0 %. Anonymized parasitologic data on positive malaria specimens examined at the Public Health Ontario Laboratories (PHOL) were also extracted from the secure biobank database, and analyzed.

### Rapid diagnostic test

Rapid diagnostic test (RDT) results for each specimen were acquired at the time of specimen processing in the clinical parasitology laboratory using the BinaxNOW Malaria RDT kit (Inverness Professional Medical Diagnostics, Scarborough, ME). Results were expressed as “positive” or “negative” at each of the *Plasmodium falciparum*-specific histidine rich protein-2 (HRP-2) and *Plasmodium* genus aldolase T2 bands, and were not quantified subjectively or objectively.

### Isolation of parasite and human DNA

200 μL of frozen whole blood from each specimen were thawed from −80 °C freezer. DNA were extracted with QIAamp DNA Mini kit (QIAGEN Sciences, Germantown, MD) using the DNA extraction protocol for blood. 60 μL of DNA were eluted for each specimen.

### Plasmodium qualitative and quantitative real time PCR

*Plasmodium* Genus and *P.falciparum/P.vivax* duplex species-specific real time PCR (qPCR) assays on 18S rRNA were performed in triplicate, and *P.malariae/P.ovale* duplex species-specific qPCR to exclude mixed infections were performed*,* as described [7, 8]. Human macro-globin qPCR were run as a positive extraction control [9]. To quantitate the 18S rRNA gene copy number for each clinical sample in the reaction, control *P. falciparum* DNA was serially diluted ranging from 11.7 million to 0.117 gene copies/reaction and was included in each run. Log gene copy numbers vs C_T_ values were plotted, and the standard curve and equation generated were used to back calculate the gene copy number for each clinical sample. Parasitemia of <0.1 % was assigned a value of 0.01 in order to determine the correlation between parasitemia with C_T_ values and copy number. Average gene copy number for each sample was calculated from *Plasmodium* genus and *P. falciparum* species-specific qPCR runs.

### Isolation of parasite and human RNA and cDNA synthesis

RNA was extracted from 200 μL of frozen whole blood using Macherey-Nagel Nucleospin RNA Blood Kit (Bethlehem, PA) with DNase digestion and eluted with 60 μL nuclease-free water. 15 μL of RNA was used for cDNA synthesis with Quanta qScript cDNA Synthesis Kit (Cat# 95047–100, Quanta Biosciences, Gaithersburg, MD).

### Asexual stages transcript expression

To determine if there was differential transcript expression between sexual and asexual stages samples, RNA transcripts of three genes were analyzed: erythrocyte binding antigen 175 (eba-175), chloroquine resistance transporter (*pfcrt*) and merozoite apical erythrocyte binding ligand (*maebl),* which were previously shown to be expressed exclusively in the asexual stages of *P. falciparum* [10–12]. Forward and reverse primers spanning exon/intron junctions to avoid any false positives on residual DNA in the RNA extracts were designed for asexual stage-specific expression erythrocyte binding antigen 175 gene (eba-175, Gene ID: 2654998) fwd 5′ GGAGGCTTTTTCAAGTATGGCCA 3′, rev 5′ CATAACTCCTTCAGAACTTTGA 3′; *Plasmodium falciparum* chloroquine resistance transporter (pfcrt, Gene ID: 2655199) fwd 5′ AAACACAGTCGTAGAGAATTGTGGTC 3′, rev 5′ AATGCGAAGGTTTTCCATGCT 3′; *maebl* (Gene ID: 811029) fwd 5′ TTTAAAAAAAAGGAATTTTCAAACATG 3′, rev 5′ GTAGCTTCTTCAAACCACTTTC 3′ and housekeeping myosin gene as extraction control (Gene ID: 813699) fwd 5′ TATCAGAGACAAAAATAAAGTTTG 3′ and rev’ 5′ CGAATCAAATAAGTCTAAATTTCG 3′. RNA transcripts were amplified with Amplitaq Gold Fast Master Mix (Life Technologies), 500nM of each primer and 2 μL of cDNA in a total volume of 20 μL. PCR conditions were 95 °C for 10 min followed by 40 cycles of 96 °C for 5 s, 60 °C (pfcrt)/58 °C (eba-175)/56 °C (maebl and myosin) for 5 s, 68 °C for 5 s with a final extension of 72 °C for 30 s. 10 μL of amplified product was visualized on 2.5 % Agarose gel at 100 V for 1 h and stained with ethidium bromide. The intensity of the eba-175 positive bands was quantitated by Quantity One software (BioRad, Hercules, CA) and percentage over background was calculated.

### Statistical analysis

Descriptive statistics were performed for all continuous and categorical parasitologic variables. Differences between categorical variables were compared using Fisher’s Exact Test. Differences between continuous variables were compared using Student’s *t*-test, or in the case of non-normally distributed variables, Mann Whitney Rank Sum test. Correlation was performed with Pearson r. Performance characteristics of eba-175 PCR were calculated using microscopy as the gold standard. Sensitivity, specificity, positive predictive value (PPV), and negative predictive value (NPV) are reported for eba-175 transcript expression in asexual parasitemia. All statistical computations were performed using Graphpad Prism 5 (Graphpad Software, Inc. La Jolla, CA). Level of significance was set at *p* < 0.05.

## Results

### Clinical samples

We identified 85 *P. falciparum* positive specimens from our de-identified malaria biobank, with storage at −80 C between August 2008 and April 2014. Forty-five specimens contained only sexual stages (gametocytes) by expert microscopy, and 40 contained only asexual stages (rings, trophozoites, schizonts) by expert microscopy. Of 45 isolated gametocytemia specimens, 34 had previously submitted specimens that were positive for isolated asexual stages, thus, the gametocytemia specimens were post-treatment in these 34 cases. For 11 specimens, no prior sample had been submitted. Parasitologic metrics are described in Table 1. All 45 isolated gametocytemia specimens had parasitemia of <0.1 %, whereas the 40 asexual-stage samples had parasitemias ranging from <0.1 to 34 %. All specimens were confirmed to contain isolate *P. falciparum* infection by species-specific qPCR.

**Table 1:** Parasitologic factors, quantitative PCR results, and RNA transcript expression in 45 *Plasmodium falciparum* positive specimens with isolated gametocytemia by expert microscopy

Sample	Microscopy	Parasitemia	HRP-2 T1	Aldolase T2	*Plasmodium* Genus Mean C_T_	StDev	*P. falciparum* Mean C_T_	StDev	Ave 18S rRNA gene copy#/rxn	eba-175	myosin
1	Gametocytes	<0.1	+	-	39.8	0.1	-	-	4	+	+
2	Gametocytes	<0.1	+	-	-	-	39.8	0.8	6	+	-
3	Gametocytes	<0.1	+	-	38.5	0.6	37.7	0.8	30	+	+
4	Gametocytes	<0.1	-	-	38.3	0.1	36.9	0.5	43	ND	ND
5	Gametocytes	<0.1	-	-	37.5	0.1	37.5	0.9	69	ND	ND
6	Gametocytes	<0.1	+	-	35.5	0.4	34.8	1.0	287	ND	ND
7	Gametocytes	<0.1	+	-	35.1	0.2	34.6	1.1	331	ND	ND
8	Gametocytes	<0.1	+	-	35.5	0.6	33.6	1.4	506	ND	ND
9	Gametocytes	<0.1	+	-	33.4	0.1	33.8	1.7	672	ND	ND
10	Gametocytes	<0.1	+	-	34.2	0.2	33.3	1.4	687	ND	ND
11	Gametocytes	<0.1	+	-	33.2	0.1	33.4	1.0	803	ND	ND
12	Gametocytes	<0.1	+	-	34.1	0.4	32.4	1.1	814	ND	ND
13	Gametocytes	<0.1	+	-	33.0	0.1	32.7	0.9	1046	ND	ND
14	Gametocytes	<0.1	+	+	33.2	0.3	32.4	0.8	1195	+	+
15	Gametocytes	<0.1	+	-	33.6	0.6	31.3	0.3	1214	-	+
16	Gametocytes	<0.1	+	-	32.5	0.4	31.7	0.5	1251	+	+
17	Gametocytes	<0.1	+	-	32.5	0.6	32.2	0.8	1422	+	+
18	Gametocytes	<0.1	+	-	33.5	0.4	31.1	0.8	1525	ND	ND
19	Gametocytes	<0.1	+	-	31.8	0.4	32.1	1.0	1754	ND	ND
20	Gametocytes	<0.1	+	-	31.6	0.3	32.1	1.2	1801	ND	ND
21	Gametocytes	<0.1	+	-	32.3	0.6	31.7	1.1	1854	ND	ND
22	Gametocytes	<0.1	+	-	31.5	0.3	31.3	0.0	1905	+	+
23	Gametocytes	<0.1	-	-	31.5	0.3	30.5	0.4	2530	-	+
24	Gametocytes	<0.1	+	-	32.2	0.8	30.9	0.8	2573	-	+
25	Gametocytes	<0.1	+	-	32.0	0.5	30.4	0.9	3267	-	+
26	Gametocytes	<0.1	-	-	30.7	0.3	31.0	1.2	3300	-	+
27	Gametocytes	<0.1	+	-	31.3	0.1	30.5	0.9	3422	ND	ND
28	Gametocytes	<0.1	+	-	30.2	0.3	29.9	0.2	4374	+	+
29	Gametocytes	<0.1	+	-	29.9	0.4	30.0	0.9	5640	ND	ND
30	Gametocytes	<0.1	+	-	30.2	0.1	29.7	0.7	5928	-	+
31	Gametocytes	<0.1	+	+	30.7	0.8	28.8	0.5	5967	+	+
32	Gametocytes	<0.1	+	+	30.0	0.1	29.3	0.8	7246	ND	ND
33	Gametocytes	<0.1	+	+	29.8	0.2	29.2	0.8	7694	-	+
34	Gametocytes	<0.1	+	+	29.6	0.2	28.9	0.9	8996	-	+
35	Gametocytes	<0.1	+	-	29.3	0.1	28.9	1.1	9686	+	+
36	Gametocytes	<0.1	+	+	28.6	0.4	29.1	1.0	10539	ND	ND
37	Gametocytes	<0.1	-	+	28.8	0.2	28.5	0.5	11110	ND	ND
38	Gametocytes	<0.1	+	+	28.4	0.7	27.5	0.5	14285	+	+
39	Gametocytes	<0.1	+	+	28.2	0.4	27.5	0.2	18236	+	+
40	Gametocytes	<0.1	+	+	27.6	0.3	27.9	1.1	20566	-	+
41	Gametocytes	<0.1	+	+	26.9	0.3	26.7	0.2	34485	-	+
42	Gametocytes	<0.1	+	-	26.7	0.2	26.4	0.6	42605	ND	ND
43	Gametocytes	<0.1	+	+	26.3	0.7	26.2	0.3	50031	-	+
44	Gametocytes	<0.1	+	+	26.5	0.1	26.0	0.8	51397	-	+
45	Gametocytes	<0.1	+	+	26.2	0.3	25.5	0.1	65208	-	+

*ND* not determined

### Rapid diagnostic test

Forty of 45 (88.9 %) isolated gametocytemia specimens, were positive for *Plasmodium falciparum*-specific histidine rich protein-2 (HRP-2; T1 band) by Binax NOW RDT (Table 1). Fourteen of 45 isolated gametocytemia specimens (31.1 %) were positive for pan-*Plasmodium* aldolase (aldolase T2 band) by Binax NOW RDT (Table 1). All 40 isolated asexual-stages specimens were positive for the HRP-2 (T1) band, and 36 out of 40 (90 %) were positive for the aldolase T2 band (Table 2).

**Table 2:** Parasitologic factors, quantitative PCR results, and RNA transcript expression in 40 *Plasmodium falciparum* positive specimens with isolated asexual-stages by expert microscopy

Sample	Microscopy	Parasitemia	HRP-2 T1	Aldolase T2	*Plasmodium* Genus Mean C_T_	StDev	*P. falciparum* Mean CT	StDev	Ave 18S rRNA gene copy#/rxn	eba-175	myosin
46	Rings	<0.1	+	-	28.8	0.9	28.8	0.4	7287	ND	ND
47	Rings, Trophs	<0.1	+	-	26.9	0.8	27.5	0.3	18727	ND	ND
48	Rings	<0.1	+	+	25.6	0.7	26.2	0.5	41458	+	+
49	Rings	<0.1	+	-	25.1	0.6	25.7	0.2	57100	ND	ND
50	Rings	<0.1	+	+	24.2	0.9	24.6	0.6	103941	ND	ND
51	Rings	<0.1	+	+	24.0	0.9	24.5	1.2	116491	+	+
52	Rings	<0.1	+	-	24.1	1.0	24.3	0.5	117324	+	+
53	Rings	<0.1	+	+	24.0	0.8	24.2	0.3	126258	+	-
54	Rings	<0.1	+	+	21.1	0.6	21.5	0.4	700623	+	+
55	Rings	<0.1	+	+	20.5	0.9	20.8	0.5	1058503	+	+
56	Rings	0.1	+	+	21.5	1.0	20.7	0.6	876634	+	+
57	Rings	0.2	+	+	23.6	1.3	23.2	0.6	209649	+	+
58	Rings	0.2	+	+	22.6	1.0	22.3	0.4	362408	ND	ND
59	Rings, Trophs	0.2	+	+	22.0	0.5	22.2	0.2	440742	ND	ND
60	Rings	0.2	+	+	21.6	1.1	20.9	0.5	798231	+	+
61	Rings, Trophs	0.3	+	+	20.9	0.7	21.2	0.2	818001	+	+
62	Rings, Trophs	0.3	+	+	20.6	0.8	20.1	0.5	1351301	+	-
63	Rings	0.4	+	+	21.2	0.8	21.8	0.6	606025	+	-
64	Rings, Trophs	0.4	+	+	21.5	1.2	21.3	0.6	684146	+	+
65	Rings	0.4	+	+	20.9	0.6	21.1	0.5	821066	+	-
66	Rings, Trophs	1.1	+	+	18.7	0.9	18.5	0.5	3761262	+	+
67	Rings	1.2	+	+	20.7	0.8	20.7	0.6	1067665	+	-
68	Rings, Trophs	1.8	+	+	19.2	1.0	19.0	0.6	2758211	ND	ND
69	Rings, Trophs, Schizonts	2.6	+	+	17.5	0.7	17.6	0.4	6849735	ND	ND
70	Rings, Trophs	3.0	+	+	19.2	0.8	19.1	0.3	2743861	ND	ND
71	Rings	3.0	+	+	17.6	0.4	18.4	0.2	5318426	+	+
72	Rings, Trophs	3.8	+	+	17.2	0.8	17.5	0.7	7901115	+	+
73	Rings, Trophs	3.8	+	+	17.2	1.0	17.2	0.4	9026304	ND	ND
74	Rings, Trophs	4.0	+	+	17.2	0.8	17.3	0.8	8628101	ND	ND
75	Rings	4.3	+	+	17.1	0.7	17.8	0.3	7438110	ND	ND
76	Rings	10.4	+	+	17.4	1.0	17.3	0.5	8171936	+	+
77	Rings	11.6	+	+	17.4	0.7	17.5	0.2	7541456	ND	ND
78	Rings	12.6	+	+	16.6	0.9	16.6	0.1	13147859	+	+
79	Rings	13.0	+	+	14.2	0.7	14.6	0.6	46984798	ND	ND
80	Rings, Trophs	14.0	+	+	14.1	0.9	14.5	0.7	50542052	ND	ND
81	Rings, Trophs	16.0	+	+	16.7	0.6	17.3	0.6	9825903	ND	ND
82	Rings	18.0	+	+	16.9	1.2	17.2	0.5	9827129	ND	ND
83	Rings	21.1	+	+	15.6	0.9	16.1	0.2	19863776	ND	ND
84	Rings	24.0	+	+	14.5	0.7	14.9	0.3	39213307	ND	ND
85	Rings, Trophs	34.0	+	+	14.4	0.5	14.9	0.5	41210046	ND	ND

*ND* not fetermined

### Quantitative real time PCR of specimens with isolated gametocytemia

Mean *Plasmodium* genus C_T_ value for 44 specimens was 31.64 ± 0.50 (median 31.6, range 26.2–39.8). One specimen was undetectable by *Plasmodium* genus qPCR but was detectable by *P. falciparum* species-specific qPCR (Sample #1, Table 1). Specimens with positive aldolase T2 bands by RDT (*N* = 14) had a mean C_T_ value of 28.63 ± 0.52 (median 28.5, range 26.2–33.2) by *Plasmodium* genus qPCR. Specimens with negative aldolase T2 bands by RDT (*N* = 30) had a mean C_T_ value of 33.05 ± 0.53 (median 32.5, range 26.7–39.8) (*p* < 0.001 compared to those with positive aldolase T2 bands) by *Plasmodium* genus qPCR. Mean *P. falciparum*-specific C_T_ value for 44 specimens was 31.04 ± 0.49 (median 31.0, range 25.5–39.8). One specimen was undetectable by *P. falciparum* species-specific qPCR but was detectable by *Plasmodium* genus qPCR (Sample #2, Table 1). Specimens with positive aldolase T2 bands by RDT (*N* = 14) had a mean C_T_ value of 28.11 ± 0.47 (median 28.2, range 25.5–32.4) by *P. falciparum*-specific qPCR. Specimens with negative aldolase T2 bands by rapid antigen test (*N* = 30) had a mean C_T_ value of 32.41 ± 0.52 (median 31.9, range 26.4–39.8) by *P. falciparum*-specific qPCR (*p* < 0.001). Mean *P. falciparum* 18S rRNA gene copy number per reaction for the 45 specimens with isolated gametocytemia was 9073.4 ± 2288.6 (median 2530.0, range 4–65208). Specimens with positive aldolase T2 bands by RDT (*N* = 14) had a mean *P. falciparum* 18S rRNA gene copy number of 21925.4 ± 5386.0 (median 12697.5, range 1195–65208). Specimens with negative aldolase T2 bands by RDT (*N* = 31) had a mean *P. falciparum* 18S rRNA gene copy number of 3269.3 ± 1365.7 (median 1422.0, range 4–42605) (*p* < 0.001).

### Quantitative real time PCR of specimens with isolated asexual stages

Parasitemia of asexual-stage specimens ranged from <0.1 to 34 % with *Plasmodium* genus C_T_ values from 14.1 to 28.8; *P. falciparum-*specific C_T_ values from 14.5 to 28.8, and 18S rRNA gene copy number from 7287 to 50,542,052 (Table 2). Log parasitemia was inversely correlated with both *Plasmodium* genus and *P. falciparum-*specific C_T_ values (Pearson r, *p* < 0.001). Furthermore, parasitemia was positively correlated with copy number (Pearson r, *p* < 0.001). Four of 40 (10 %) specimens were negative for the aldose band on RDT, and all had low parasitemia of <0.1 %. Specimens with positive aldolase T2 band by RDT had mean *Plasmodium* genus C_T_ value of 19.31 ± 0.52 (median 19.2, range 14.1–25.6) and mean *P. falciparum-*specific C_T_ value of 19.46 ± 0.51 (median 19.1, range 14.5–26.2). Specimens with negative aldolase T2 band by RDT had mean *Plasmodium* genus C_T_ value of 26.23 ± 1.04 (median 26, range 24.1–28.8) and mean *P. falciparum-*specific C_T_ value of 26.58 ± 0.99 (median 26.6, range 24.3–28.8) (*p* < 0.001 vs those with a positive aldolase T2 band). Mean 18S rRNA gene copy number for specimens with positive aldolase T2 bands by RDT was 8,637,126 ± 2,285,803 (median 2,751,036, range 41,458–50,542,052) and for specimens with negative aldolase T2 bands by RDT was 50,109.5 ± 24,808.1 (median 37,914, range 7287–117,324) (*p* < 0.001). Higher asexual parasitemia was also associated with positivity of the aldolase T2 band, with 6 out of 10 (60 %) of <0.1 % parasitemia being aldolase T2 band-positive, compared to 30 out of 30 (100 %) specimens with ≥0.1 % parasitemia being aldolase T2 band-positive (Fisher’s Exact Test, *p* = 0.002).

### Expression of asexual stage-specific transcripts

**Fig. 1:**
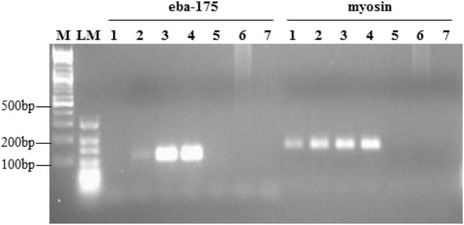
Eba-175 and myosin transcript expression. M, DNA Ladder; LM, Low Range DNA Ladder; 1 and 2, isolated *P. falciparum* gametocytemia cDNA (sample # 30 and 35); 3 and 4, asexual *P. falciparum* parasitemia cDNA (sample # 60 and 66); 5, human cDNA; 6, *P. falciparum* DNA; 7, blank negative control

An initial analysis of 3 asexual and 3 gametocytemia specimens at all three genes showed no amplification of eba-175 on all 3 gametocytemia samples whereas *pfcrt* and *maebl* showed 3 and 2 amplifications respectively (data not shown) and thus eba-175 was chosen for further analysis. Due to the limited number of primary specimens available, a subset of 25 isolated gametocytemia and 20 asexual-stages specimens were analyzed. All 20 (100 %) asexual stages specimens, and 12 out of 25 (48 %) isolated gametocytemia specimens demonstrated eba-175 expression (Tables 1 and 2; Fig. 1). To ensure there was no cross-reactivity of human cDNA and *P. falciparum* DNA, 2 human cDNA and 8 *P. falciparum* DNA extracts were tested with both eba-175 and myosin PCR and no cross-reactivity was observed. Using microscopy as the gold standard, eba-175 PCR demonstrated 100 % sensitivity and 52 % specificity for asexual parasitemia, with a PPV of 62.5 % and NPV of 100 % (Table 3). The mean band intensity in isolated gametocytemia specimens was 107 % ± 2.6 % (median 104 %, range 101–133 %) and in asexual-stage specimens was 147 % ± 9.3 % (median 134 %, range 101–216 %). Thus, there was higher eba-175 transcript expression in asexual-stage specimens than in isolated gametocytemia specimens (Mann Whitney, *p* < 0.001). The mean age of individuals with eba-175 positive specimens was 37 years (range 16–61 years) versus 44 years (range 2–64 years) for eba-175 negative specimens, and these were not significantly different.

**Table 3:** Performance of *Plasmodium falciparum* eba-175 transcript expression compared to gold-standard microscopy for identification of asexual parasitemia

eba-175 transcript expression	Microscopy		Predictive values
	Isolated asexual stages (N)	Isolated sexual stages (Gametocytes) (N)	
eba-175 Positive	20	12	PPV^a^ = 62.5 %
eba-175 Negative	0	13	NPV^b^ = 100 %
Performance Characteristics	Sensitivity^c^ = 100 %	Specificity^d^ = 52 %	

^a^positive predictive value for asexual parasitemia, calculated as (true positives)/(true positives + false positives)

^b^negative predictive value for asexual parasitemia, calculated as (true negatives)/(true negatives + false negatives)

^c^sensitivity for asexual parasitemia, calculated as (true positives)/(true positives + false negatives)

^d^specificity for asexual parasitemia, calculated as (true negatives)/(true negatives + false positives)

## Discussion

Malaria is a potentially life-threatening imported infection, with 1,687 cases diagnosed in the United States in 2012, the majority of which occurred in those traveling to visit friends and relatives [13]. Malaria is an illness of particular public health importance as it is vector-borne, and has the potential to emerge in North America with expansion of the range of *Anopheles* vectors [14], and increasing importation from the tropics, particularly by certain high-risk types of travelers who may be semi-immune with asymptomatic parasitemia [15, 16]. Given that malaria is life-threatening, potentially emergent, and of public health importance, it is of great relevance to the health of North Americans. Asexual stages (i.e., rings, trophozoites and schizonts) are clinically relevant stages, whereas sexual stages (i.e., gametocytes) are clinically irrelevant to the human host, meaning that they do not require treatment for resolution of malaria. Gametocytes, however can be taken up by *Anopheles* mosquitoes via a blood meal, thus perpetuating the *Plasmodium* life cycle. In this regard, gametocytemia is relevant from a public health and disease transmission perspective, but has little relevance to the clinician caring for an ill patient, or the laboratorian communicating clinically relevant results back to the care team.

Microscopy remains the gold-standard diagnostic test for malaria, but with ongoing attrition of microscopy expertise in clinical laboratories across North America, hospitals and labs are increasingly reliant on non-parasitologic testing methods such as RDT and molecular assays. However, these tests cannot reliably differentiate asexual from sexual parasitemia, and are thereby limited in their clinical performance characteristics, both for new diagnoses of malaria, and for following response to treatment. Both RDT and PCR can remain positive long after successful treatment of malaria, and resolution of clinical symptoms [17]. At present, use of PCR is fairly restricted to reference laboratories, though PCR plays an increasingly important role in quality control of both microscopy and RDT, and in investigating febrile returned travelers at high risk of malaria, but with negative smear and RDT results [18]. Thus, defining and optimizing the best possible diagnostic strategies for malaria, and the life stages of *Plasmodium* should be a priority.

We have demonstrated that the BinaxNOW commercial RDT and a validated qPCR based on the *Plasmodium* 18S rRNA region were unable to differentiate asexual from sexual stages of *P. falciparum*, however, the presence of the pan-*Plasmodium* aldolase T2 band was correlated to both higher parasitemia by microscopy and 18S rRNA gene copy number. This latter finding is consistent with previous research in which the absence of the aldolase T2 band served as a reasonable basis on which to rule out high parasitemia [19]. Although RNA transcript expression of eba-175 had a low positive predictive value (62.5 %) in this study, it provided a negative predictive value of 100 % for asexual stages, suggesting that it is potentially a good marker to rule-out asexual parasitemia in specimens received at the laboratory. Band intensity of eba-175 transcript expression was also greater in specimens containing asexual stages by microscopy. The low specificity (52 %) and positive predictive value observed in this study may be explained by sub-microscopic level of asexual stages, or any residual eba-175 transcripts leftover from prior asexual stages in the isolated gametocytemia specimens. It is known that both symptomatic and asymptomatic individuals may carry sub-microscopic infections with *P. falciparum*. Moreover, sub-microscopic infections have been reported to be persistent and produce gametocytes. As microscopy and RDT are limited in their ability to detect these infections, especially in endemic populations, PCR assays have the potential to further detect sub-microscopic parasitemia [18, 20]. Furthermore, it may be possible that residual transcripts from asexual stages remain in the circulation during a patient’s recovery. In an analysis comparing microscopy, RDT and qPCR results, we found that the 18S rRNA DNA can circulate in the bloodstream even after parasites are no longer observed in Giemsa-stained blood smears (Boggild unpubl. data). Given this observation, slow clearance of the eba-175 transcript may also be a possibility.

Microscopy is the gold standard for diagnosis of malaria, and identification of the different life stages is critical to provision of appropriate treatment. For instance, a patient who continues to have fever while on malaria treatment or thereafter will be managed differently if asexual parasitemia continues compared to isolated sexual-stage parasitemia. Expert microscopy demands a high level of training and skill, and in malaria non-endemic countries, this expertise is a challenge to maintain; hence, an increasing reliance on RDT and molecular assays, which may not provide adequate differentiation of asexual from sexual stages during the investigation of a febrile returned traveler, or during follow-up parasitemia while the patient is on anti-malarial treatment. As microscopy expertise is lost over time in malaria non-endemic areas, PCR and RDT are being increasingly used for malaria confirmation. In those circumstances, eba-175’s high NPV sufficiently excludes asexual parasitemia in a specimen that may be positive for HRP-2 antigenemia by RDT and possibly for 18S rRNA,or some other molecular target, by PCR. Thus, the ultimate goal should not be to confirm the presence of gametocytes, but rather, the absence of asexual stages such as rings and trophozoites. Further research into markers of isolated asexual or sexual parasitemia, whether RNA transcripts or expressed proteins, that provide both high positive and negative predictive values is warranted.

Several limitations of this analysis should be acknowledged. Our small sample size reflected the availability of specimens, and the limited volume of primary samples available for RNA transcript analysis. However, we included all specimens in our biobank with isolated gametocytes during the inclusion period of August 2008 to April 2014 in this study. In future, a larger sample size with time-series analysis and clinical linkage will more rigorously identify the role of eba-175 transcript expression in the diagnosis and follow-up of malaria. Such an analysis will become more important as microscopy expertise in clinical laboratories continues to wane. Second, the low specificity and PPV of eba-175 for asexual specific-stages limits our ability to use this target as a rule-in test. On the other hand, we demonstrated 100 % NPV for exclusion of asexual stages, which reinforces the utility of eba-175 as a “rule-out” test for clinically relevant stages of *P. falciparum*.

## Conclusions

Asexual stage-specific eba-175 RNA transcript expression provided reasonable negative predictive value for exclusion of asexual parasitemia in clinical samples, but was present in both isolated gametocytemia and asexual stage specimens.

## Abbreviations

C_T_: Cycle threshold
eba: Erythrocyte binding antigen
HRP2: Histidine rich protein 2
NPV: Negative predictive value
PCR: Polymerase chain reaction
PPV: Positive predictive value
qPCR: Quantitative real time polymerase chain reaction
RDT: Rapid diagnostic test
RNA: Ribonucleic acid
rRNA: Ribosomal ribonucleic acid
